# Left atrial strain and recurrence of atrial fibrillation after thoracoscopic surgical ablation: a subanalysis of the AFACT study

**DOI:** 10.1007/s10554-022-02645-5

**Published:** 2022-07-20

**Authors:** Sarah W. E. Baalman, Nicoline W. E. van den Berg, Jolien Neefs, Wouter R. Berger, Eva R. Meulendijks, Rianne H. A. C. M. de Bruin-Bon, Berto J. Bouma, Wim Jan P. van Boven, Antoine H. G. Driessen, Joris R. de Groot

**Affiliations:** 1 Amsterdam UMC, Department of Cardiology, Location University of Amsterdam, Room B2-254, Meibergdreef 9, 1105 AZ, PO Box 22700, Amsterdam, The Netherlands; 2 Amsterdam UMC, Department of Cardiothoracic Surgery, Location University of Amsterdam, Amsterdam, The Netherlands; 3 Amsterdam Cardiovascular Sciences, Heart Failure & Arrhythmias, Amsterdam, The Netherlands

**Keywords:** Atrial fibrillation, Thoracoscopic surgical abation, Mini-maze, Left atrial strain, Mechanical dispersion

## Abstract

To assess transthoracic echocardiographic (TTE) left atrial (LA) strain parameters and their association with atrial fibrillation (AF) recurrence after thoracoscopic surgical ablation (SA) in patients in sinus rhythm (SR) or in AF at baseline. Patients participating in the Atrial Fibrillation Ablation and Autonomic Modulation via Thoracoscopic Surgery trial were included. All patients underwent thoracoscopic pulmonary vein isolation with LA appendage exclusion and were randomized to ganglion plexus (GP) or no GP ablation. In TTEs performed before surgery, LA strain and mechanical dispersion (MD) of the LA reservoir and conduit phase in all patients, and of the contraction phase in patients in SR were obtained. Recurrence of AF was defined as any documented atrial tachyarrhythmia lasting > 30 s during one year of follow-up. Two hundred and four patients (58.6 ± 7.8 years, 73% male, 57% persistent AF) were included. At baseline TTE 121 (59%) were in SR and 83 (41%) had AF. Patients with AF recurrence had lower LA strain of the reservoir phase (13.0% vs. 16.6%; *p *=  < 0.001) and a less decrease in strain of the conduit phase (−9.0% vs. −11.8%; *p *= 0.006), regardless of rhythm. MD of the conduit phase was larger in patients with AF recurrence (79.4 vs. 43.5 ms; *p *= 0.012). Multivariate cox regression analysis demonstrated solely an association between LA strain of the reservoir phase and AF recurrence in patients in SR (HR 0.95, *p *= 0.046) or with AF (HR 0.90, *p *= 0.038). A reduction in LA strain of the reservoir phase prior to SA predicts recurrence of AF in both patients with SR or AF. Left atrial strain assessment may therefore add to a better patient selection for SA.

## Introduction

Catheter ablation (CA) or thoracoscopic surgical ablation (SA) have been shown an effective treatment option to restore sinus rhythm (SR), and is recommended in patients with symptomatic atrial fibrillation (AF) refractory to antiarrhythmic drugs [1, 2]. Thoracoscopic surgical ablation in particular, may be a more effective treatment strategy in patients with advanced AF or failed prior CA [1, 3]. Absence of AF recurrence within 2-years after SA varies from 70 to 80% [4, 5], with a low 30-days complication rate [6]. However, the generally more extensive left atrial (LA) lesions than applied during CA may negatively affect the LA function, leading to an iatrogenic decrease in LA function that may itself affect the risk of AF recurrence [4, 7]. Therefore, identifying patients who may or may not benefit from SA in terms of AF recurrence remains important, especially as risk scores or validated outcome predictors for this procedure are lacking.

Left atrial strain reflects the dynamic aspect of the LA and thereby may better reflect LA remodeling than conventional echocardiographic parameters [12, 13]. Fibrosis of the LA wall has proven to be inversely related to LA strain and strain rate [14]. As atrial remodeling is a predictor for AF recurrence and echocardiography is easily applicable, LA strain has been shown an interesting parameter to predict recurrence of AF. LA strain and LA strain derived mechanical dispersion (MD) have been associated with AF recurrence after CA, even in the absence of any other echocardiographic abnormality [7–11]. Most studies to date have only included patients in SR at time of echocardiography, and data involving strain analysis in patients with AF during echocardiography are scarce. Hypothetically, this is because of the presumed incoherency of the different atrial phases during a cardiac cycle during AF, and thereby the lack of the specific strain patterns including a reservoir, conduit and contraction phase as can be determined in patients in normal SR [15]. However, particularly in patient cohorts with advanced AF, which is usually persistent AF (perAF) enlarged left atria and/or previously failed CA, patients tend to be more often in AF than in SR during echocardiography before the procedure. Therefore, the aim of this study is to assess I) the association between LA strain parameters and AF recurrence in patients who underwent SA, II) if strain can also be used as a predictor of AF recurrence in patients with AF during baseline echocardiography and III) how LA strain differs between patients who are in SR or with AF during baseline echocardiography.

## Materials and methods

### Patient population

This study is a sub-analysis of the Atrial Fibrillation Ablation and Autonomic Modulation via Thoracoscopic Surgery (AFACT) trial that investigated epicardial ganglion plexus (GP) ablation during SA for advanced AF [4]. The study was approved by the local ethical committee and all patients provided written informed consent. The outline of the study and main results have been described in detail previously [4]. In short, included patients (*n *= 240) underwent SA with left atrial appendage (LAA) exclusion and were randomized to additional ablation of the ganglion plexus (GP) or no additional GP ablation. The study population included both paroxysmal AF (pAF) and persistent AF (perAF) patients. Extensive inclusion and exclusion criteria are described in the main paper of the AFACT trial [4]. All patients underwent thoracoscopic pulmonary vein ablation with a bipolar clamp (Isolator Synergy clamp, Atricure, West Chester, Ohio). In patients with perAF, additional LA ablation lines consisting of a roof and trigone line and conforming to the Dallas lesion set were made [4, 16]. All patients were followed-up every three months for one year with a clinical visit, ECG and 24-h Holter monitoring, in accordance with the 2017 HRS/EHRA/ECAS Expert consensus statement [17]. Suspected patients for AF recurrence were encouraged to obtain additional rhythm monitoring. Recurrences of AF were defined as any documented atrial tachyarrhythmia lasting more than 30 s on any rhythm recording or a standard ECG recording showing AF. Antiarrhythmic drugs (AAD) were discontinued after 3 months, and only reintroduced in case of AF recurrence. The first three months after the procedure were regarded as blanking period for outcome analysis. As the AFACT trial demonstrated no difference in AF recurrence between the randomized treatment groups, data of patients with and without GP ablation were pooled for this analysis.

### Echocardiography

All 240 patients underwent two-dimensional transthoracic echocardiography (TTE) before the procedure (2D-TTE) (Vivid 9 machine, GE VingmedUltrasound AS, Horten, Norway). All TTE examinations were performed within one month before SA and specifically assessed LA volume and function. Four- and two chamber views were obtained by experienced cardiac echocardiographists according to the recommendations of the American Society of Echocardiography [18]. Recordings were made using a 1.6-MHz to 3.2-MHz transducer (System 9; GE Healthcare, Milwaukee, WI), digitized, and analyzed offline with EchoPAC (GE Healtcare, 2015 General Electronic Co.). The LA myocardial wall was traced manually in each four chamber view suitable for strain analysis. Hereafter, the software created a region of interest automatically that we adjusted and segmented manually to the anatomy of the LA. After correcting the region of interest, the software calculated the different strain parameters as stated below. The average frame rate of the images was 51.9 ± 8 frames per second. The strain analysis protocol detailed below was implemented both in patients who were in SR at the time of echocardiography as in those with AF, notwithstanding the expected inability to measure LA strain of the contraction phase of patients with AF [15].

### Left atrial strain and mechanical dispersion

**Fig. 1:**
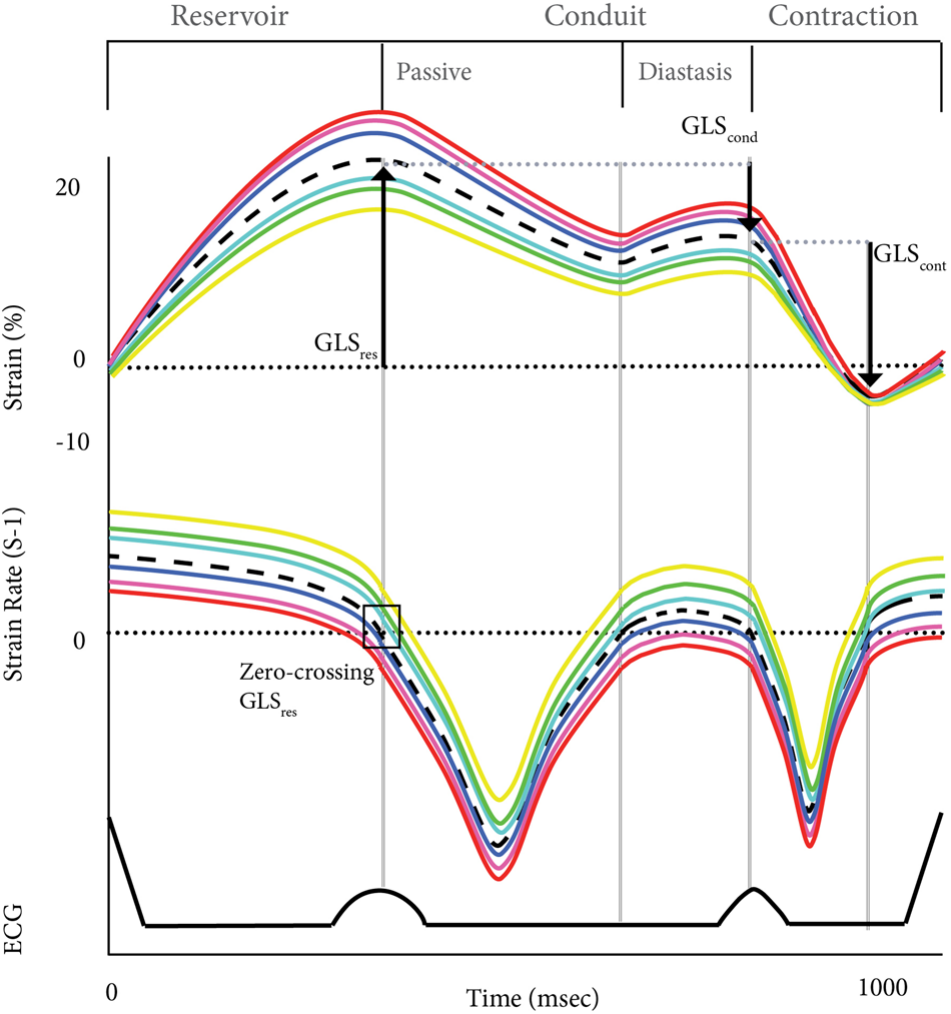
Global longitudinal strain and segmental strain. Global longitudinal strain (GLS) (dotted line) and strain of the six different segments (lateral segments: yellow & green, roof segment: light blue & purple, septal segments: blue & red). The strain values of the three different LA phases are determined by using the recommendations of the EACVI/ASE/Industry Task Force to standardize deformation imaging and the zero crossing points of the strain rate curve as reference. GLS_res_ = GLS reservoir. GLS_cond_ = GLS conduit, GLS_cont_ = GLS contraction

The following strain and dispersion parameters were obtained from the baseline TTE: LA longitudinal global strain (GLS) and segmental strain of the LA reservoir (GLS_reservoir_), conduit (GLS_conduit_), and contraction phase (GLS_contraction_) as stated in the EACVI/ASE/Industry Task Force to standardize deformation imaging [15]. (Fig. 1), wherein: GLS_reservoir_: peak strain during the reservoir phase (positive value). GLS_conduit_: decrease in strain at the end of the conduit phase (negative value). Note that, in patients with AF at time of TTE, GLS_conduit_ has the same value as GLS_reservoir_, however with a negative sign. GLS_contraction_: maximum decrease in strain during the contraction phase, relative to the end of the conduit phase. This was only measured in patients in SR during TTE (negative value).

**Fig. 2:**
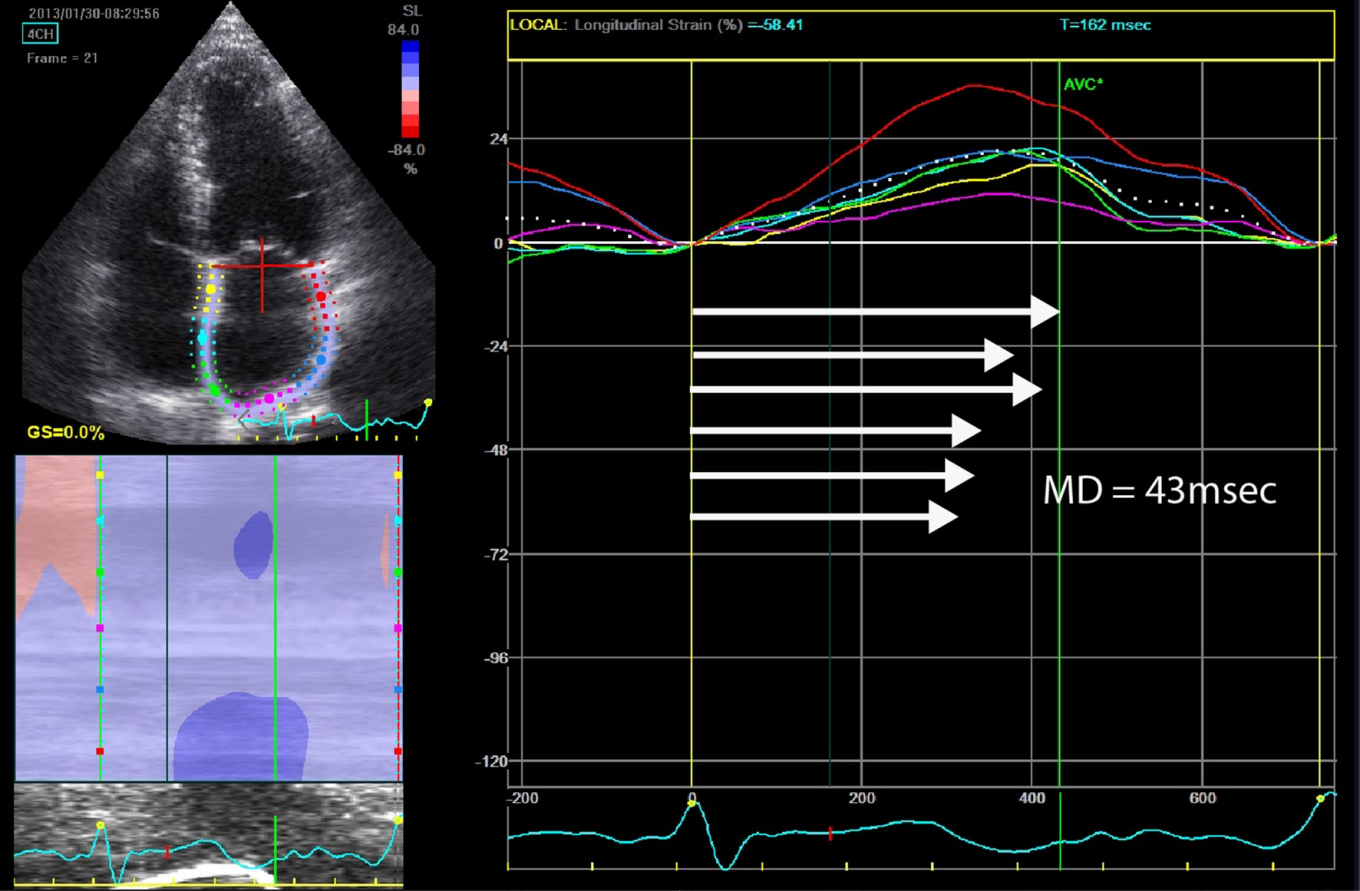
Mechanical dispersion. Mechanical dispersion in milliseconds calculated from the 6 different colored curves representing the different segments of the myocardial wall of the left atrium. MD = mechanical dispersion. Msec = milliseconds

Strain parameters were determined from an apical four chamber view using the zero crossing points in the strain rate curve as reference (Fig. 1) using speckle tracking echocardiography. Only images where the LA segments could be properly traced were included. We documented heart rate (HR), and manually confirmed the cycle length in milliseconds (msec). A complete strain pattern (GLS_complete_) was defined as a strain curve in which all data points needed for analysis of the three phases could be identified (Fig. 1). This was only determined in patients in SR during TTE, as strain of the contraction phase cannot be determined in AF. An incomplete strain pattern (GLS_incomplete_) was defined as a pattern in which not all phases could be determined in patients in SR. The strain values in patients with GLS_incomplete_ that could be obtained were still taken into account in the final analysis. Mechanical dispersion (MD) was defined as the standard deviation (SD) of the time to peak maximum or minimum strain of the 6 different LA segments in the three LA phases, corrected for cycle length (Fig. 2), wherein: MD_reservoir_ : SD of the time to peak reservoir strain (GLS_reservoir_) of the 6 different segments, measured in all patients and in patients in SR or with AF separately. MD_conduit_: SD of the time of the 6 different segments to reach the end of the conduit phase (GLS_conduit_), only measured in patients in SR during TTE. MD_contraction_: SD of the time to peak minimum strain during the contraction phase (GLS_contraction_), only measured in patients in SR during TTE.

### Statistical analyses

Data were analyzed using SPSS statistics version 23.0 (IBM Corporation, New York, USA) and R version 3.6.2 (R foundation, Vienna, Austria). All analyses, except strain analyses of the contraction phase and MD analysis of the conduit and contraction phase, were performed for the entire group and for patients in SR and with AF separately. Continuous variables were expressed as mea*n *± (SD), or median (IQR) and categorical variables as absolute numbers or percentages. For the comparison of variables between two independent groups, independent sample t-test or Mann–Whitney U test was used. The Multivariate Cox proportional models were used to determine the impact of LA strain (%) and MD (per 10 ms on AF recurrence after SA expressed as hazard ratios (HR). All clinical parameters associated with AF recurrence with a *p*-value < 0.05 in the univariate analysis were included in the multivariate analysis. For the survival analysis we used the median of each variable as cut off point. Kaplan–Meier (KM) curves were used to present time to first AF recurrence of significant predictors of AF recurrence. We determined the difference in survival distributions for the two curves by using the log-rank test. A two-sided *p*-value < 0.05 was considered to be significant in all tests.

## Results

### Study population

A total of 204 (85%) of the 240 patients were included in the current analysis. In 23 patients the TTE did not conform to the criteria needed for strain analysis. An additional 13 patients had to be excluded because of lost to FU (*n *= 9) or death (*n *= 4), which precluded outcome analysis at one year of follow-up. Patient characteristics of the included patients are shown in Table 1. The mean age of all patients was 59 ± 8 years, 54 (27%) were female and 116 (57%) patients had perAF.

**Table 1:** Patient characteristics

	All (*n *= 204)	AF (*n *= 83)	SR ( *n *= 121)	P- value
Patient characteristics				
Female (%)	54 (27%)	18 (22%)	36 (30%)	0.262
Age	58.6 ± 7.8	59.0 ± 8.4	58.0 ± 7.5	0.575
BMI, kg/m^2^	27.5 ± 3.9	27.6 ± 4.0	27.4 ± 3.8	0.781
Persistent AF	116 (57%)	72 (87%)	44 (36%)	< 0.001
Previous cath. PVI (%)	50 (24%)	17 (21%)	33 (27%)	0.503
Stroke	16 (8%)	6 (7%)	10 (8%)	0.996
Risk factors				
Hypertension	86 (42%)	39 (47%)	47 (39%)	0.311
CHF	8 (4%)	4 (5%)	4 (3%)	0.718
DM	12 (6%)	3 (4%)	9 (7%)	0.402
VD	20 (10%)	7 (8%)	13 (11%)	0.760
Hyperlipidemia	90 (28%)	39 (30%)	51 (27%)	0.814
Medication				
ACE inhibitors	44 (22%)	23 (28%)	20 (16%)	0.117
Beta-blockers	106 (52%)	52 (63%)	54 (45%)	0.047
Laboratories				
Creatinine, μmol/l	84.0 (20.2)	86.0 (17.5)	82.0 (19.0)	0.014
CRP, mg/l	1.3 (2.2)	1.5 (2.4)	1.1 (2.2)	0.628
NT- proBNP, pmol/l	242.0 (444.3)	515.0 (544.7)	136.5 (162.8)	< 0.001
TSH, mE/l	2.0 (1.6)	2.0 (1.5)	1.9 (1.5)	0.596

Patients characteristics of all 204 patients, and patients in SR or with AF during baseline TTE separately. Values are mean ± SD, n (%), or median (IQR), unless otherwise indicated. BMI = Body mass index, CHF = Chronic heart failure, DM = Diabetes mellitus, VD = Vascular disease, PVI = Pulmonary vein isolation, CR*P *= C-reactive protein, NT-proBN*P *= N-terminal prohormone of brain natriuretic peptide, TSH = Thyroid-stimulating hormone

#### Echocardiography

A total of 83 (41%) patients had AF and 121 (59%) were in SR at the time of baseline TTE. Significantly more perAF than pAF patients had AF at the time of TTE (87% vs. 36%; *p *=  < 0.001). Patients with AF during TTE used beta-blockers more often (63% vs. 45%; *p *= 0.047), had higher NT-proBNP (515.0 pmol/l vs. 136.5 pmol/l; *p *< 0.001) and creatinine concentration (86.0 μmol/l vs. 82.0 μmol/l; *p *= 0.014).

#### AF recurrence

Twenty-nine (29%) patients experienced AF recurrence within one year after SA (with exclusion of the first three months blanking period). Plasma NT-proBNP concentration was the only clinical parameter that differed between patients with and without AF recurrence (378.0 pmol/l vs. 210.0 pmol/l; *p *= 0.008). There was no significant difference in the proportion of perAF on outcome (68% vs. 52%; *p *= 0.064). The proportion of patients randomized to GP ablation was similar in the group with and without AF recurrence [4].

### Left atrial strain and mechanical dispersion

Echocardiographic parameters of all patients at the time of TTE as well as patients with SR or AF separately are displayed in Table 2. In 96 (73%) out of the 121 patients in SR, a complete strain pattern could be obtained, in the other patients one or more strain parameters could not be measured. Only GLS of the reservoir phase was measured in 100% of the 204 included patients. Patients with AF at the time of baseline TTE had a larger indexed left atrial volume (LAVI) (44.2 ml/m^2^ (IQR 13.4) vs. 37.6 ml/m^2^ (IQR 15.3) *p *= 0.007), lower GLS_reservoir_ (8.8% (IQR 7.2) vs. 21.4% (IQR 11.1) *p *< 0.001), GLS_conduit_ (−8.8% (IQR 7.2) vs. −11.0% (IQR 8.5) *p *< 0.001) phase and increased MD_reservoir_ (88.9 ms (IQR 109.0) vs. 47.2 ms(IQR 64.6) *p *< 0.001) than patients in SR.

**Table 2:** Baseline echocardiography

	All (n = 204)	AF (n = 83)	SR ( n**= 121)**	P- value	N (%)
LA structure/function					
LA diameter, mm	42 ± 5.6	43.5 ± 5.4	41 ± 5.6	0.002	204 (100%)
LAVI, ml/m^2^	40.7 (15.1)	44.2 (13.4)	37.6 (15.3)	0.007	203 (99.5%)
GLS_reservoir_, %	15.2 (13.3)	8.8 (7.2)	21.4 (11.1)	< 0.001	204 (100%)
GLS_conduit_D_, %	− 9.8 (7.8)	− 8.8 (7.2)	− 11.0 (8.5)	< 0.001	179 (87.7%)
GLS_contraction_, %	− 10.2 (5.7)	NA	− 10.2 (5.7)	NA	96 (47.1%)
Dyssynchrony					
GLS_Complete_	96 (73.3%)	NA	96 (73.3%)	NA	121 (59.3%)
MD_reservoir_, msec	59.4 (84.2)	88.9 (109.0)	47.2 (64.6)	< 0.001	195 (95.6%)
MD_conduit_D_, msec	48.3 (60.2)	NA	48.3 (60.2)	NA	108 (52.9%)
MD_contraction_, msec	14.7 (31.8)	NA	14.7 (31.8)	NA	85 (41.7%)

Echocardiographic parameters of all 204 patients, and patients in SR or with AF during baseline TTE separately. Values are mean ± SD, n (%), or median (IQR), unless otherwise indicated. LAVI = left atrial volume index, GLS = global longitudinal strain, MD = mechanical dispersion, msec = milliseconds

#### Strain parameters associated with AF recurrence

Table 3 shows the difference in echocardiographic parameters on AF recurrence during FU regardless of the rhythm during TTE. Patients with AF recurrence had lower GLS of the reservoir phase (13.0% (IQR 10.2) vs. 16.6% (IQR 13.6), *p *< 0.001) and a smaller decrease in strain during the conduit (−9.0% ± 5.3 vs. −11.8% ± 7.5, *p *= 0.006) and contraction phase (only patients in SR) (−9.3% ± 4.4 vs. −11.8% ± 6.0 *p *= 0.046) than patients without AF recurrence. Mechanical dispersion during all three phases was larger in patients with- than in patients without AF recurrence. Only the MD_conduit_ was significantly increased in patients with AF recurrence compared to patients without AF recurrence (79.4 ms (IQR 78.4) vs. 43.5 ms (IQR 53.8), *p *= 0.012).

**Table 3:** Echocardiography and recurrence of atrial fibrillation

	All (n= 204)	AF rec (n= 59)	No AF rec (n = 145)	P- value	N
LA structure/function					
LA diameter, mm	42.0 ± 5.6	42.6 ± 5.3	41.8 ± 5.7	0.328	204 (100%)
LAVI, ml/m^2^	40.7 (15.1)	44.6 (14.4)	38.2 (14.7)	0.002	203 (99.5%)
GLS_reservoir_, %	15.2 (13.3)	13.0 (10.2)	16.6 (13.6)	< 0.001	204 (100%)
GLS_conduit_D_, %	− 9.8 (7.8)	− 9.0 ± 5.3	− 11.8 ± 7.5	0.006	179 (87.7%)
GLS_contraction_, %	− 10.2 (5.7)	− 9.3 ± 4.4	− 11.8 ± 6.0	0.046	96 (47.1%)
Dyssynchrony					
GLS_Complete_	96 (73.3%)	19 (67.9%)	77 (82.8%)	0.148	121 (59.3%)
MD_reservoir_, msec	59.4 (84.2)	70.5 (110.1)	56.4 (76.9)	0.366	195 (95.6%)
MD_conduit_D_, msec	48.3 (60.2)	79.4 (78.4)	43.5 (53.8)	0.012	108 (52.9%)
MD_contraction_, msec	14.7 (31.8)	20.8 (54.7)	13.1 (25.6)	0.069	85 (41.7%)

Echocardiographic parameters of all 204 patients, and patients with or without AF recurrence after SA. Values are mean ± SD, n (%), or median (interquartile range), unless otherwise indicated. LAVI = left atrial volume index, GLS = global longitudinal strain, MD = mechanical dispersion, msec = milliseconds

In univariate analysis lower GLS strain of the reservoir phase (HR 0.95 (CI 95%: 0.92–0.98) *p *< 0.001) and a smaller decrease in strain during the conduit phase (HR 1.08 (CI 95%: 1.02–1.14) *p *= 0.010) were associated with AF recurrence analyzing all patients regardless of the rhythm. When the cohort was analyzed for patients in AF or SR respectively at the time of TTE, GLS_reservoir_ (HR 0.96 (CI 95%: 0.91–1.00) *p *= 0.038) and MD_conduit_ (HR 1.04 per 10-ms increase (CI 95%: 1.01–1.08) *p *= 0.022) remained significantly associated with absence of AF in patients in SR, whereas, GLS_reservoir_ (HR 0.92 (CI 95%: 0.85–1.00) *p *= 0.040) and GLS_conduit_ (HR 1.09 (CI 95%: 1.00–1.18) *p *= 0.040) remained significantly associated with AF recurrence in patients with AF at the time of TTE.

**Fig. 3:**
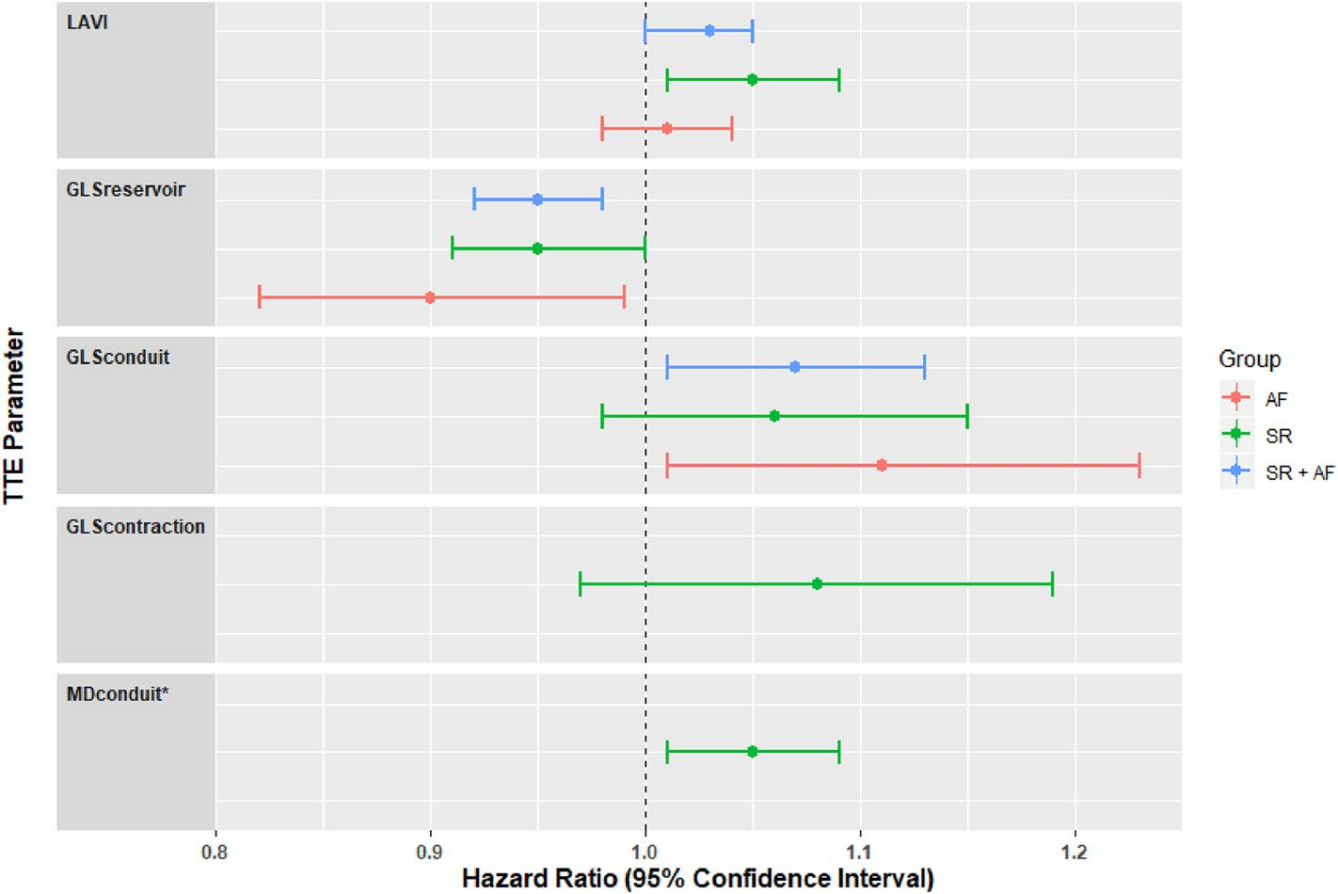
Multivariate analysis. A forest plot showing the hazard ratio and 95% confidence interval associated with LA strain variables in predicting AF recurrence after SA. All clinical parameters with *p*-value < 0.05 in the univariate analysis (AF type, previous PVI, hypertension) were included in the multivariate model. LAVI = left atrial volume index, GLS_reservoir_ = Global longitudinal strain of the reservoir phase, GLS_conduit_ = Global longitudinal strain of the conduit phase, GLS_contraction_ = Global longitudinal strain of the contraction phase, MD_conduit_ = Mechanical dispersion of the conduit phase. Blue bar = entire population (SR + AF), Green bar = patients in SR during baseline TTE, Red bar = patients in AF during baseline TTE.* Hazard Ratios per 10-msec increase

The following clinical variables with a *p*-value < 0.05 in univariate analysis were included in the multivariate model: AF type, hypertension and previous CA. In the multivariate analysis as shown in Fig. 3, GLS_reservoir_ remained significantly associated with AF recurrence in the entire group (HR 0.95 (CI 95%: 0.92–0.98) *p *= 0.005) and in both groups of patients in SR (HR 0.95 (CI 95%: 0.91–1.00) *p *= 0.046) or with AF (HR 0.90 (CI 95%: 0.82–0.90) *p *= 0.038). GLS of the conduit phase remained significantly associated with AF recurrence in the entire group (HR 1.07 (CI 95%: 1.01–1.13) *p *= 0.028) and in patients with AF separately (HR 1.11 (CI 95%: 1.01–1.23) *p *= 0.038). Despite a numerically similar HR in patients in SR during baseline TTE, the association between GLS_conduit_ and AF recurrence did not reach statistical significance. Mechanical dispersion of the conduit phase (HR per 10-ms increase 1.05 (CI 95%: 1.01–1.09) *p *= 0.017) remained an independent predictor of AF freedom after SA in patients in SR.

### Predictors of atrial fibrillation recurrence in patients with AF or SR during TTE

**Fig. 4:**
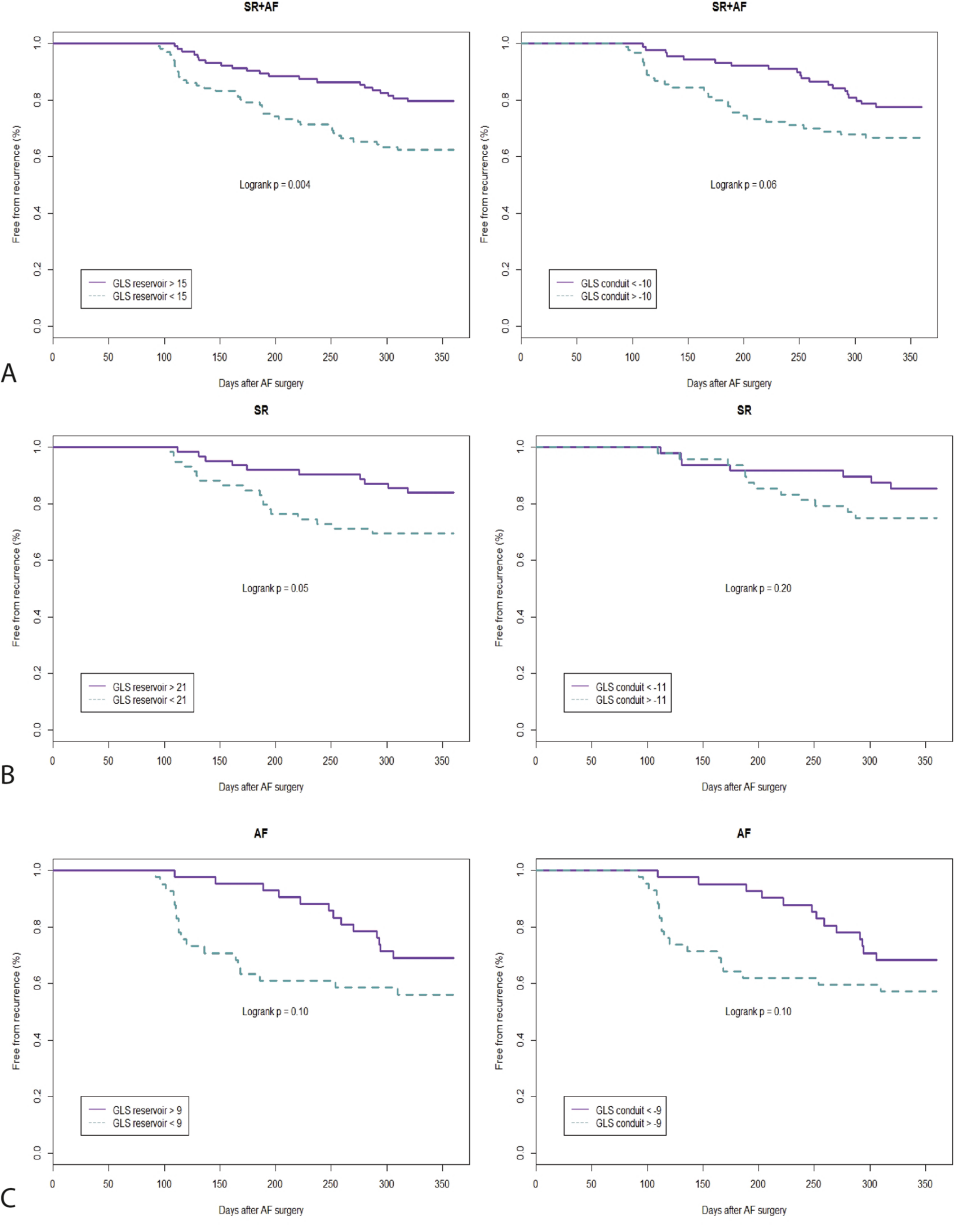
Survival analysis. Kaplan Meier curves of strain of the reservoir (GLS_reservoir_) and conduit (GLS_conduit_) phase of A) the entire population (AF + SR). B) Patients in SR during baseline echocardiography. C) Patients with AF during baseline echocardiography. GLS = Global longitudinal strain. MD = Mechanical dispersion

As GLS of the reservoir and conduit phase were independently associated with AF recurrence, we determined whether these parameters accurately predict AF recurrence over time in all patients and patients with AF and SR separately. Figure 4 shows KM curves of GLS_reservoir_ and GLS_conduit_ of all patients (3A), patients in SR (3B) and patients with AF (3C) at the time of baseline TTE. AF free survival was significantly higher in patients with a high GLS_reservoir_ (GLS_reservoir_ > 15%, *p *= 0.004). The magnitude of the difference in GLS_reservoir_ or GLS_conduit_ in patients with and without AF recurrence was similar in patients who were in SR and patients with AF at the time of TTE (Fig. 4B and C), although not statistically significant.

## Discussion

This sub-analysis of the randomized AFACT trial shows that LA strain parameters determined on baseline echocardiography are associated with AF recurrence in patients who undergo SA for advanced AF [4]. Our most important findings are that: (1) There is a significant difference in LA strain and MD values between patients with AF and SR during echocardiography before SA. (2) Both GLS and MD measured at baseline echocardiography are associated with recurrence of AF after SA. (3) Global longitudinal strain of the reservoir phase is a predictor of AF recurrence in patients in SR or with AF during baseline echocardiography.

### LA strain and AF recurrence after SA

Other studies have shown the predictive value of strain to AF recurrence in patients in SR and AF undergoing CA [19]. However, most often patients with AF at the time of echocardiography were not included in these studies. To the best of our knowledge, this is the first study that combines patients in SR and AF and investigates these subgroups separately during baseline echocardiography in relation to AF recurrence after SA.

Our results show a difference in LA strain between patients in SR and with AF at the time of TTE, including a difference in MD. Besides the presence of AF during TTE which may have caused a more chaotic activation and contraction pattern, AF type (i.e. persistent or paroxysmal), may have contributed to this difference. The majority of patients with AF during TTE were indeed perAF patients, with expectedly a more advanced atrial remodeling (e.g. increased LAVI and fibrosis) compared to pAF patients.

An inverse relationship between LA strain and quantity of fibrosis assessed by late gadolinium enhancement CMR has been described. Patients with a high grade of fibrosis and high burden of AF do have lower strain values [14]. This correlation between LA strain and fibrosis may point at a more advanced atrial fibrotic substrate and may be as such, in part, responsible for the difference in strain between patients with or without AF recurrence. Of note, in the AFACT study all perAF patients received more extensive LA ablation with additional ablation lines, which may have affected LA function during follow-up but not at baseline TTE [4]. As scarring is associated with a decline in LA systolic function and therefore negatively affects LA strain [20], we cannot exclude that more rigorous ablation may eventually lead to further decline of the LA systolic function, and may have impacted on the risk of AF recurrence in patients with a low GLS_reservoir_ at baseline. LAA exclusion is not expected to influence LA function as a prior retrospective group comparison, reports a decrease in LA reservoir and conduit strain after SA without an impaired LA contraction function, irrespectively of LAA exclusion [21].

Previous studies have reported GLS of the reservoir phase as an independent predictor of AF recurrence after CA [7–9, 11, 13, 22]. Our study confirms the hypothesis that baseline GLS_reservoir_ is also a predictorof AF recurrence after SA. Interestingly, we extend this previous observation by showing that this relation remains not only in patients in SR during echocardiography but also in patients with AF. Multivariate analysis performed in this study showed that LA strain may be a more precise predictor of AF recurrence after SA than LAVI, as it significantly predicts AF recurrence in patients with AF during baseline echocardiography besides patients in SR.

### Mechanical dispersion and AF recurrence

In line with GLS strain, MD was associated with AF recurrence. MD reflects heterogeneity of the left ventricular or LA myocardial contraction and can accurately quantify the difference in timing and regional function [23, 24]. There are reports linking MD of the left ventricle (LV) with the occurrence of ventricular arrhythmias [25]. Less information is available of MD of the LA in association with AF recurrence after CA or SA [22]. The current study is concordant with previous findings associating MD with AF recurrence. However, a significant relation was only demonstrated in patients in SR during TTE. The incoherent pattern of atrial activation of the different segments during AF may have rendered MD futile in relation to AF recurrence in patients with AF during echocardiography. In addition, it is impossible to measure MD of the conduit phase of patients with AF as there is no start of the contraction phase in these patients.

### Clinical implications

Strain analysis, in particularly left ventricle strain, has been extensively studied, and has proven to be a valuable tool in cardiovascular medicine [26, 27].

Left ventricular strain is readily implemented in clinical practice, but LA strain hold the potential for a clinical role too [28]. Strain measurements directly reflect the transformation of contracting myocardium and may therefore reflect LA remodeling. Indeed, the LA mechanical function may reflect the atrial fibrotic substrate and thereby the chance of a successful procedure outcome. Thereby, LA strain and possibly MD may add to decision making models to improve risk stratification and patient selection for invasive treatment for AF. Our study shows that LA strain as a screening parameter is promising in patients with SR but equally useful in patients with AF. This is relevant, as these patients are at particular risk for AF recurrence, irrespective of the type of intervention. Our results show a higher risk of AF recurrence in patients with a GLS_reservoir_ < 15% regardless of rhythm in our population. Our analysis shows that dichotomic assessment of strain parameters provides useful prognostic information. Future work may lead to distinct cut-off values that could be used in patient in SR or with AF during baseline TTE for risk stratification of AF recurrence after CA or SA. It may be too early to substitute LAVI or any other parameter with LA strain, however using LA strain may be an additional echocardiographic parameter of interest, irrespective of rhythm at the time of echocardiography.

## Limitations

Several limitations should be acknowledged. First, the used software to measure LA strain was initially designed to analyze strain of the LV. The different anatomy of the left atrium may have complicated measuring LA strain, especially in this specific population wherein a divergent left atrium is not uncommon. We do, however, show that these software packages can be used as well for LA strain analysis. The use of tracking 6 segments for LA strain assessment may be not as accurate as strain assessment of the LV, the LA has several regions (i.e. interatrial septum, ostia of the pulmonary veins and LAA) that cannot contract or reflect atrial deformation. Also, measuring LA strain is more time consuming than other echocardiographic parameters. We therefore believe that software designed for LA strain is needed to gain more accurate results, especially if we want to implement LA strain into clinical practice. Indeed, some vendors now include specific LA strain software in their analysis packages. The AFACT trial was not powered for the current sub-analysis, therefore, the relatively low number of patients, particularly those in AF, may have affected the statistical significance of our findings.

## Conclusions

A reduction in LA strain of the reservoir phase is associated with AF recurrence after SA in both patients in SR or with AF during baseline echocardiography. LA strain may add to a better patient selection for invasive treatment for AF.
